# The rose and the name: the unresolved debate on biotechnological terms

**DOI:** 10.1111/1751-7915.13522

**Published:** 2019-12-24

**Authors:** Martí Domínguez, Juli Peretó, Manuel Porcar

**Affiliations:** ^1^ Language Theory and Communication Sciences Department University of Valencia Valencia Spain; ^2^ Institute for Integrative Systems Biology I^2^SysBio (University of Valencia – CSIC) Paterna Spain; ^3^ Department of Biochemistry and Molecular Biology University of Valencia Burjassot Spain; ^4^ Darwin Bioprospecting Excellence SL Paterna Spain

## Abstract

The largest survey on the perception of synthetic biology‐related disciplines (Porcar et al., 2019,*EMBO Rep* 20) recently revealed that the Spanish society does not have a very positive perception of the term synthetic biology. On the other hand, the terms *biotechnology* and even *genetic engineering* received relatively higher scores. The issue of nomenclature and perception is a classical one in science perception studies. Synthetic biologists have been debating their neologism (Synthetic Biology, from now on SB) for years. Even in a 2006 blog, Rob Carlson discussed the various labels for the new field, such as *intentional biology*, *constructive biology*, *natural engineering*, *synthetic genomics* and *biological engineering*. This diversity of names, along with the above mentioned negative public perception of the term synthetic biology, raises the question on whether the term itself is suitable or whether it could, in an extreme scenario, be replaced by another combining scientific consensus with public acceptance.

## Graphical abstract

Our work describes the perception on SynBio by the community of practitioners and even co‐founders of the East Coast of the USA.

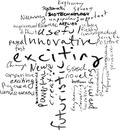

What's in a name? that which we call a rose

By any other name would smell as sweet.

William Shakespeare's *Romeo and Juliet* (Act 2, scene 2 )

The term *synthetic biology* was coined in 1910 by the French biophysicist Stéphane Leduc, and it was used as the title of one of his books in 1912 (Leduc, 1912; Peretó, 2016). Leduc sought to achieve the synthesis of artificial life ‘by directing the physical forces which are its cause’ (cited in Keller, 2002) in the best mechanist tradition (Peretó, 2016). Leduc practiced ‘synthetic biology’ as a logical extension of ‘synthetic chemistry’; therefore, the origin of this name should be associated with the search of strictly material mechanisms and components to explain life. Typically, Leduc and other anti‐vitalistic scientists searched for non‐biological chemical mixtures producing systems that not only mimicked living processes but that could also generate real living matter. In this way, the adjective ‘synthetic’ refers to this blend of chemistry and physics associated with the origin of life, taking us back to a forgotten episode of the biological explanations (Keller, 2002).

While the name is old, the field in its modern form only emerged about less than twenty years ago (Lorenzo and Danchin, 2008; Porcar and Peretó, 2014). At the beginning of the 21st century, scientists at MIT, especially those working with pioneers Tom Knight and Drew Endy, among others, started talking about a new discipline, which they called synthetic biology (Endy, 2005; Andrianantoandro *et al.*, 2006; McLeod and Nerlich, 2017). Then, new journals, conferences, as well as funding agencies, started to use the term SB. However, there is an ongoing (and behind‐the‐scene) debate stimulated by the difficulties encountered by biotechnologists when communicating their work to society. In this context, terms such as *synthetic*, *modified*, *manipulated*, *unnatural*, or even *gene* or *genetically* are received very negatively during scientific talks addressed to general audiences. Nonetheless, it must be stressed that overstatements by scientists (or their institutional press offices) and the use of misleading metaphors or headlines in the media might also have a negative effect on the general acceptance of some terms (Porcar and Peretó, 2018; Porcar *et al* 2019). In short, the question that arises from this discussion is whether an ‘inappropriate’ scientific name represents a disadvantage in the growth of a discipline or, as Shakespeare said, a rose by any other name ‘would smell as sweet’.

With the aim of answering this question, we have used a top‐down approach to explore the perception of SB from the perspective of its own practitioners and – in some cases – co‐founders. In particular, we have conducted a qualitative survey among biotechnological researchers working in the East Coast area of the USA, namely in research centres in the Boston area. These include Harvard Medical School (Boston), Harvard University (Cambridge) and MIT (Cambridge), institutions that are known to have very relevant teams working on several fields related to SB. In order to carry out the survey, a questionnaire with 22 items under the title ‘What’s in a name?’ was designed. The questions related to SB were as follows: ‘Do you think it is a good name? Yes/No/(Dk/Da)’, ‘If not, why?’, ‘What are your feelings regarding SB? Refer them using 3 adjectives’, ‘Do you think there is a more suitable name? If you do, propose a new name’, ‘Do you think it is too late to change it? Please explain briefly your answer’. These questions were followed by other queries about GMOs, cloning and CRISPR, with the same questions as in SB about the adequacy of the name. At the end of the survey, we asked if ‘an inappropriate scientific name can be a problem for research’. Prior to carrying out the survey, and in order to detect any errors (i.e. presence of biases, unclear sentences, phrasing problems or misunderstandings), the questionnaire was distributed to two independent experts (Victor de Lorenzo, from CNB‐CSIC, Madrid, Spain, and Kristie Tanner, from Darwin Bioprospecting Excellence SL, Valencia, Spain), who did not find any confusing phrasing or possible misinterpretation.

Surveys were conducted in July 2019 by one of the authors (MD), who requested a personal appointment with researchers working in this field in the Boston area. Each participant was requested to fill in the survey by hand (Fig. 1), and the personal information collected included their full name, professional status, their affiliation (faculty or research centre) and their age. A total of 27 surveys were collected, which can be considered a significant number taking into account the difficulties associated with gathering participants that are relevant researchers in the SB research area. We interviewed the following scientists (listed in alphabetical order by last name: all explicitly agreed to be named in this manuscript): Cristina Agapakis (Ginkgo Bioworks); Simone Bruno (MIT); George Church (Harvard Medical School); Saurja Dasgupta (Massachusetts General Hospital); Daniel Duzdevich (Massachusetts General Hospital); Kevin Esvelt (MIT); Kevin Fox (MIT); Theodore Grunberg (MIT); Jeremy Gunawardena (Harvard Medical School); Hsinho Huang (MIT); Jennifer Kaczmarek (MIT); Suhyun Kim (Harvard Medical School); Mihir P. Khambete (MIT); Elizabeth Libby (Harvard Medical School); Wesley L. Marques (MIT); Cynthia Ni (MIT); Juan Pérez‐Mercader (Harvard University); Kristala L. Jones Prather (MIT); Yili Qian (MIT); Randy Rettberg (iGEM); Pamela Silver (Harvard Medical School); Jack W. Szostak (Harvard University); Mike Veling (Harvard Medical School); and Jeffrey C. Way (Harvard Medical School). Three other interviews conducted with MIT researchers were done; however, they chose to appear anonymously in the article (their opinions are reported as MIT1, MIT2 and MIT3). Only two of the surveys (Pamela Silver and MIT2) were completed via email. In order to determine the most common adjectives used by the respondents to refer to each concept (SB, GMOs, cloning and CRISPR), the qualitative analysis software NVivo 12 was used.

**Figure 1:**
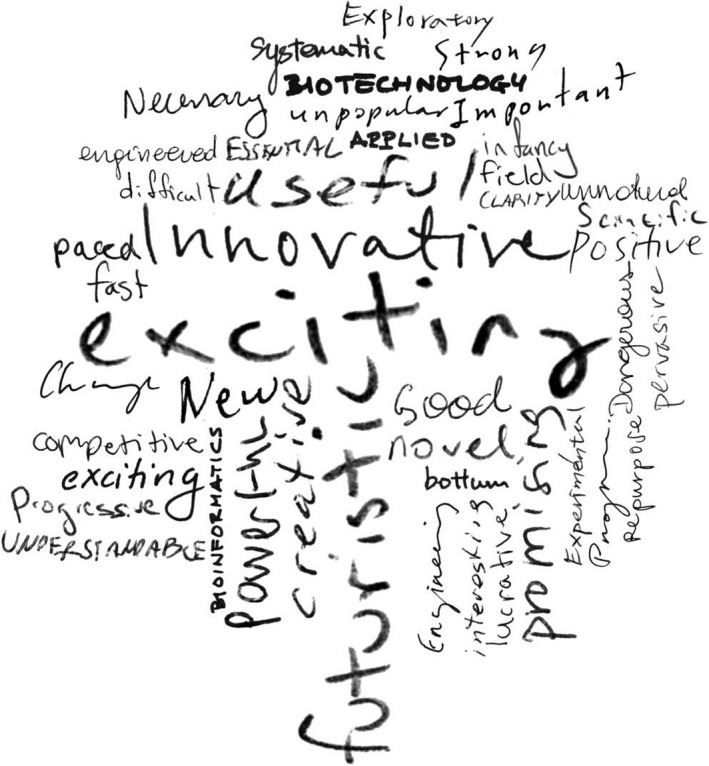
Word cloud collecting handwritten adjectives used to refer to the term synthetic biology.

A total of 63 % of the surveyed scientists answered that SB is a ‘good name’ (Fig. 2A), although almost 40 % either disagreed or chose the DK/Da option. Therefore, there is a significant percentage of researchers working on SB who do not explicitly support the term. For instance, Elizabeth Libby considers that it is not a good name because ‘it fails to emphasize that it draws from naturally occurring systems’. Similarly, for Suhyun Kim ‘Synthetic biology is distinguished by its philosophy: an engineering mindset that aims to build new things using biology, or create new biology itself.’, and Daniel Duzdevich rhetorically asks: ‘It’s not really “synthetic”, is it? It’s just cellular biology’. Regarding the feelings stirred up by the term SB, almost all the adjectives showed a positive perception. The most common were as follows: *exciting*, *novel*, *futuristic* and *useful* (Fig. 3A). In this sense, only two out of the 61 adjectives had a clearly negative connotation (*dangerous* and *lucrative*). In general, though, scientists working with SB have a very enthusiastic opinion about their discipline, which they qualify as *promising*, *creative* and *essential*. Very few scientists proposed a new potential name for it: MIT1 and MIT2 suggested *biological engineering*, Kristala Prather chose *constructive biology*, George Church preferred *biomolecular engineering* and Randy Rettberg recommended using *friendly biology*.

**Figure 2:**
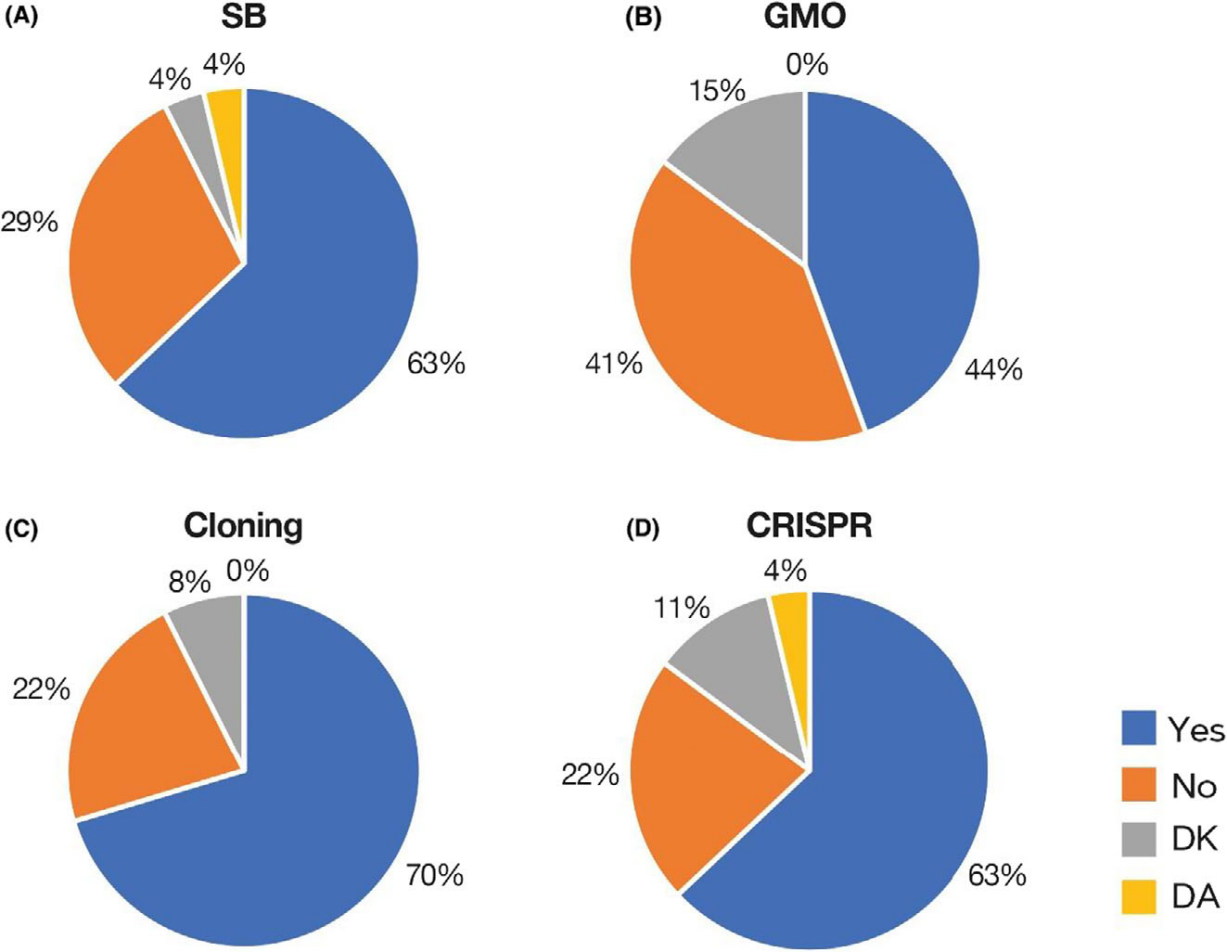
Pie charts showing the acceptance of several biotechnological terms: (A) synthetic biology, (B) GMO, (C) cloning and (D) CRISPR.

**Figure 3:**
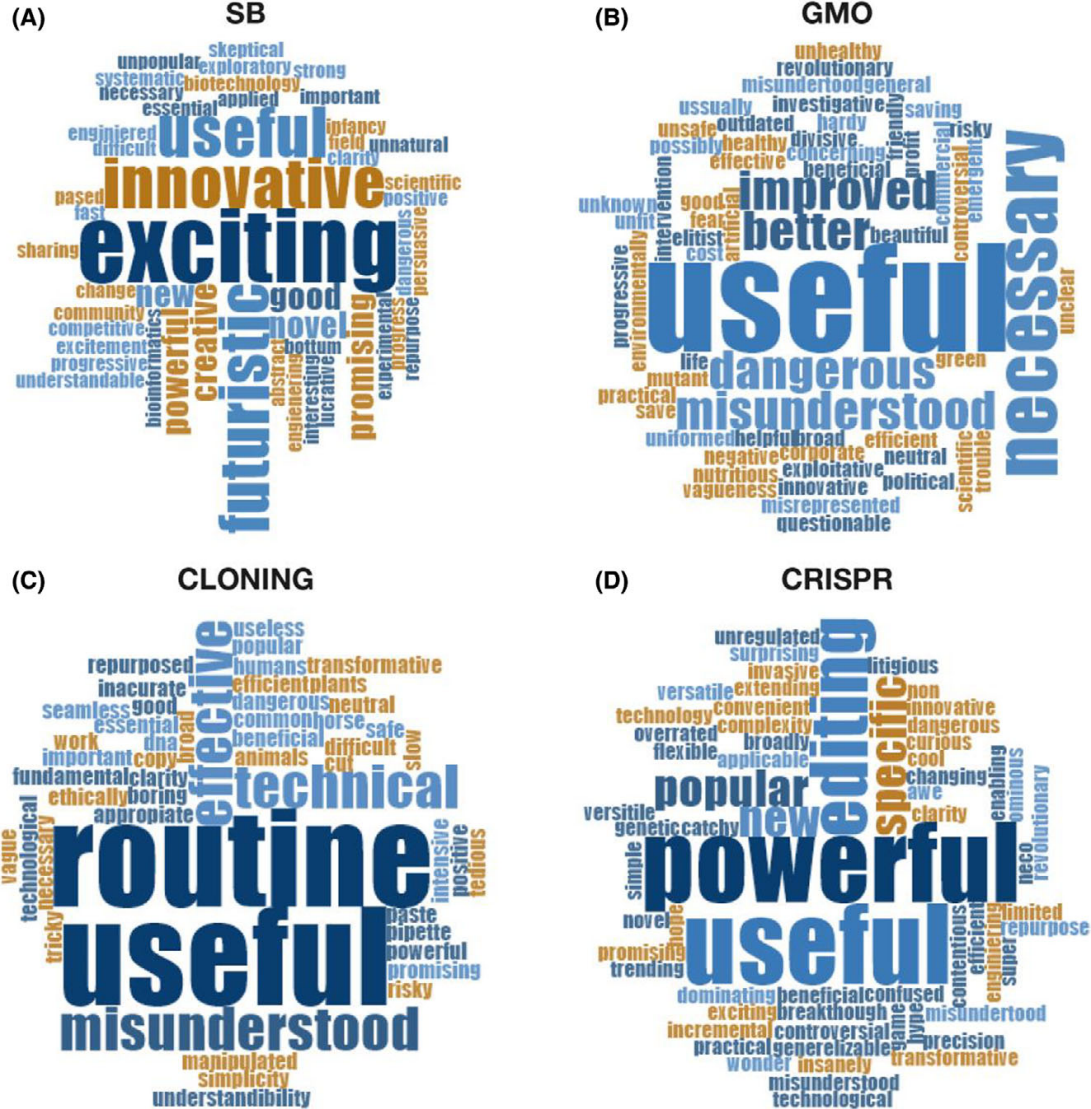
Word cloud including adjectives used to qualify several biotechnological terms: (A) synthetic biology, (B) GMO, (C) cloning and (D) CRISPR.

A large fraction of the participants (63 %) stressed that SB is a suitable name and/or it is too late to change: ‘The name is already very widely used, and is a simple, concise and acute description’ Jack Szostak answered; for Jeffrey C. Way, ‘the underlying technology is the problem, not the name’; and for Kristala Prather, ‘the term has strong penetration in science and would be practically impossible to change’. Pamela Silver also agreed that it seems that it is now too late to change it ‘because there are a number of centres, national and international programs, and training programs. If someone charismatic changed the name and redefined it, then that could happen’. On the other hand, for Kevin Esvelt it is just a matter of time: ‘Sufficient data or negative perceptions will discourage use’, and Suhyun Kim thinks that ‘it is an early field and [the name] can change’. MIT2 agreed this could happen: ‘The field currently described by the term will evolve. The two distinctive approaches to biological engineering originally associated with the term “synthetic biology” will be of declining relevance to researchers, funders, regulators and civil society as methods advance’.

In the case of GMOs, 44% of the surveyed researchers answered that it was a good name, whereas 41% answered that it was not (with 15% of Da/Dk answers) (Fig. 2B). Elizabeth Libby explained that it was not a good name because it ‘creates fear and distrust of “unnatural” things’. The adjectives were not as enthusiastic as with SB, and in some cases expressed ‘fear’, describing GMOs as *dangerous* or *unsafe*. However, the most abundant adjective was *useful* (Fig. 3B). The most commonly suggested name was *genetically improved* (GI), but researchers also proposed terms such as *bioengineered organisms* (Suhyun Kim), *edit & transgenic* (George Church) or *artificially bred* (MIT3). George Church believes that the current name is no longer suitable (‘It was okay, but became a target’). Regarding the possibility of changing the name, almost half of the scientists surveyed (41 %) thought that it is already too late (‘it is a well‐known name, for better or worse’, says Cynthia Ni; ‘It’s now a loaded term’ answers Pamela Silver), and even that it could be risky: ‘GMO is well‐established in language. I think a change would cause the public to feel that scientists were trying to “trick them”’ (Kristala Prather). However, George Church and Randy Rettberg are of the opinion that it is not too late to change it (‘No. Just do it’, Rettberg answered), and Elizabeth Libby replied that it ‘depends on context: there is already a lot of mistrust in direct to consumer products and crops’.

The results for ‘cloning’ show that 70% of the surveyed scientists think it is a good name, while 22 % do not, with a very reduced percentage of Da/Dk (Fig. 2C). The feelings concerning cloning were also a mixture of negative and positive perceptions. After *useful*, the most common adjectives were *routine* and *dangerous* (*risky*) (Fig. 3C). In one case, the respondent specified ‘Not in humans’ (Pérez‐Mercader). Cloning is seen by scientists as a powerful biotechnological tool, necessary and useful, but also one with clear risks. In general, researchers avoided proposing a new possible name. Only Mike Veling suggested one: *DNA manipulation*. Jeffrey C. Way answered that *cloning* was not a good name because it ‘refers to the process of growing up’ and Randy Rettberg explained that ‘as we do it more we will need more specific names’, because ‘it is too broad: cloning corn vs. cloning babies’. And he added: ‘It is used for both making new people and inserting a piece of DNA into a Phasmid’. Regarding the possibility to change it, there was a disparity of opinions and a significant percentage of experts think it is not too late to find a better name (37%). In this sense, Cynthia Ni suggests it could be changed because ‘the term cloning in molecular biology is only used by people in the field and could be changed if everyone agreed to it’ and Mike Veling thinks that ‘the general public doesn't think of the word “cloning” in the same way as the scientific community’. However, for Kristala Prather, it is too late to change it because ‘this word goes back many decades now and covers a wide range of technologies’.

The results for CRISPR were quite similar to those collected for the term cloning: 63 % of positive answers and 22 % of negative answers, respectively, with a reduced percentage of Da/Dk (Fig. 2D). Nevertheless, adjectives to define CRISPR were very positive. The most used ones were *powerful*, followed by *useful* and other optimistic terms such as *hope*, *awe*, *wonder* or *breakthrough* (Fig. 3D). There were also some adjectives reflecting doubt or scepticism (i.e. *overrated*) or deception (i.e. *misunderstood*). Very few alternative names were proposed: *DNA editing* (George Church) *directed genetic therapy* (Juan Pérez‐Mercader) and *genome editing* (Randy Rettberg). Regarding the possibility of changing the term, there was almost a tie between the surveyed scientists (52% think it is too late to change it). For Jack Szostak it is ‘widely used and easy to say’, Jeffrey C. Way does not find ‘reasons to change’, and Pamela Silver remarked that ‘there will be much more technology in this space over the coming years’. MIT3 thought it was too late ‘after “CRISPR baby” was in the news’. However, for Kristala Prather, ‘CRISPR is an acronym, so it has a deeper meaning’ and it could be changed because ‘it is the newest of the words and thus less embedded’. For Jennifer Kaczmarek and Kevin Fox, Prather’s graduate students, it is not too late because term is confusing and ‘I don’t understand the concept’ (JK), and ‘People who don't frequently use CRISPR might not have the knowledge of how it works, and the name itself does not feel descriptive enough to give any sort of better understanding. This is particularly true since the acronym is so prevalent but the meaning of the acronym itself is not’ (KF). For George Church, it is not too late because ‘Editing predates CRISPR’ and ‘it doesn’t describe the technology (none of the six letters of the acronym apply)’. Finally, Randy Rettberg was confident that it will be changed: ‘we will change when we have more techniques’.

A large number of participants (20 out of the 27 surveyed scientists) agreed that an inappropriate scientific name could be a problem for research (Duzdevich adds: ‘Language matters’). However, Randy Rettberg answered that ‘rarely: look at the quark names’, and Pamela Silver pointed out the potential antagonism between disciplines: ‘Mainly when a new area infuses jealousy into the established investigator framework. This happened in the early days of recombinant DNA’. Jack Szostak expressed this clarification: ‘Can be if it creates or become associated with negative public perceptions, but it is usually not the fault of the name *per se*’. In the same way, Kristala Prather expressed her hesitation: ‘Yes, [it could be a problem for research], especially if it causes confusion regarding its use and/or purpose’.

The main goal of this qualitative survey was to gather the opinions of SB practitioners on scientific terminology associated with SB. In short, does a name help to build a subject/discipline or, conversely, can it be a huge problem, as in the case of GMOs? Several articles have clearly shown how GMOs bad reception is due to an awful public communication (Sjöberg, 2004; Qin and Brown, 2007). The (negative) perception of GMOs has been used as a model to evaluate and anticipate public reaction to other emergent technologies, like nanotechnology (Sylvester *et al.*, 2009; Brunel *et al.*, 2018). In this sense, a similar (negative) reaction to SB or CRISPR may arise in the future, so it is imperative to set up a concretion scenario and a dialogue with society, including agents, policymakers, media and the general public, in order to create consensus on what is useful for the society and what is not (Porcar and Peretó, 2018).

Also the purpose of this research was to explore whether more accurate scientific terminology could reduce the bad perception of some disciplines. In this sense, Jeffrey C. Way expressed his doubts in a note warning about the possibility ‘to manipulate people with word‐games instead of being honest’. Certainly, it could be true that the use of more euphemistic or even more metaphoric/suggestive names to avoid saying that something has been altered or modified by biotechnological means (in short, that a certain product is ‘unnatural’) could trick or mislead the public.

Word games or otherwise, what this survey presents is an unresolved debate regarding biotechnological naming. A meaningful public understanding of biotechnological terms is crucial to improve the social perception and to allow scientists to work with less restrictive legislation (this could be especially true in Europe). Paraphrasing Shakespeare, what’s in a biotechnological name? That which we call synthetic biology by any other name would be… better understood?

## Conflict of interest

None declared.
